# A case of dermatomyositis in a patient with central core disease: unusual association with autoimmunity and genetic muscle disease

**DOI:** 10.1186/s12969-021-00598-y

**Published:** 2021-06-30

**Authors:** Min Jung Kim, Mi Hyeon Kim, Sung-Hye Park, Yeong Wook Song

**Affiliations:** 1 Division of Rheumatology, Department of Internal Medicine, Seoul Metropolitan Government-Seoul National University Hospital Boramae Medical Center, Seoul, South Korea; 2 Division of Rheumatology, Department of Internal Medicine, Seoul National University Hospital, Daehak-ro, Jongno-gu, Seoul, 03080 South Korea; 3 Department of Pathology, Seoul National University College of Medicine, Seoul, South Korea; 4 Medical Research Center, Institute of Human-Environment Interface Biology, Seoul, South Korea

**Keywords:** Dermatomyositis, Central core disease, Congenital myopathy

## Abstract

**Background:**

Dermatomyositis is an inflammatory muscle disease caused by immune-mediated muscle injury, and central core disease (CCD) is a congenital myopathy associated with disturbed intracellular calcium homeostasis and excitation-contraction coupling. To date, CCD has not been reported to have autoantibodies or coexist with inflammatory myopathy.

**Case presentation:**

Here, we described the case of a 25-year-old woman who had progressive proximal muscle weakness, myalgia, pruritic macular rash, skin ulcers, and calcinosis. Dermatomyositis was initially suspected based on the clinical symptoms accompanied by elevated muscle enzyme levels, electromyography abnormalities, and a positive antinuclear antibody test. However, the patient’s muscle biopsy revealed the characteristic findings of both dermatomyositis and CCD, suggesting that dermatomyositis occurred in this patient with previously asymptomatic CCD. The patient did not have any pathogenic gene mutations associated with congenital myopathy, including *RYR1* and *SEPN1* in targeted next-generation sequencing. She received high-dose glucocorticoid therapy and azathioprine with a significant improvement in muscle strength.

**Conclusions:**

We present a case of rare coexistence of dermatomyositis and CCD. Clinicians should be aware that patients with CCD may have inflammatory myopathy that responds well to immunosuppressive therapy.

## Background

Dermatomyositis and central core disease (CCD) are well-recognized, distinct muscle diseases. Dermatomyositis is an autoimmune inflammatory myopathy associated with progressive proximal muscle weakness, a characteristic rash and extramuscular manifestations [1]. CCD is a congenital myopathy characterized by the early onset of hypotonia, predominantly proximal muscle weakness and histopathological features of focally reduced oxidative enzyme activity seen as the central core. Congenital dislocation of the hips and scoliosis and foot deformities are common in CCD [2]. CCD is mainly due to dominant mutations in the ryanodine receptor 1 (*RYR1*) gene, which encodes channel proteins involved in skeletal muscle calcium homeostasis and excitation-contraction coupling (ECC) [3]. The severity of muscle weakness in patients with CCD due to dominant *RYR1* mutation is usually mild; most patients with CCD achieve independent ambulation with slow progression, and are clinically asymptomatic even in about 40% of patients with histological signs of disease [4, 5].

There seems to be little relationship between inflammatory myopathy and genetic muscle diseases. However, there have been descriptions of patients with muscular dystrophy and myositis-specific autoantibodies [6]. The presence of autoantibodies may exacerbate muscle inflammation due to the high levels of autoantigens expressed by regenerating muscle cells, leading to overlapping inflammatory myopathy. To date, congenital myopathy has not been reported to have autoantibodies or coexist with inflammatory myopathy.

Here, we report a rare case of dermatomyositis coexisting with CCD presenting as severe progressive proximal muscle weakness and a skin rash with ulcerations and calcinosis.

## Case presentation

A previously healthy 25-year-old Korean woman had a two-month history of progressive proximal muscle weakness. She had myalgia, fever, severe alopecia and a pruritic macular rash over her face, hand dorsum, upper anterior chest and upper back. She had difficulty in sitting up and could only move around using a wheelchair. She had no hypotonia, feeding difficulty, dysarthria, motor developmental delay, or orthopaedic abnormalities in infancy or early childhood. Her family history was unremarkable.

**Fig. 1:**
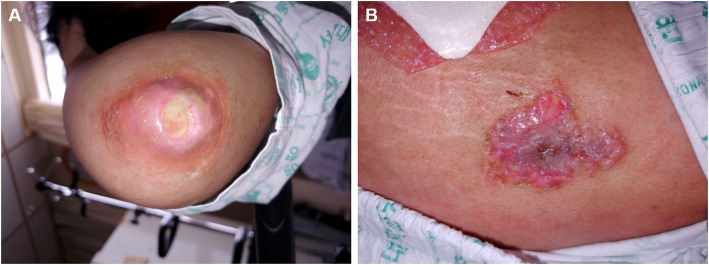
Skin ulcerations on **A** the elbow and **B** thigh

**Fig. 2:**
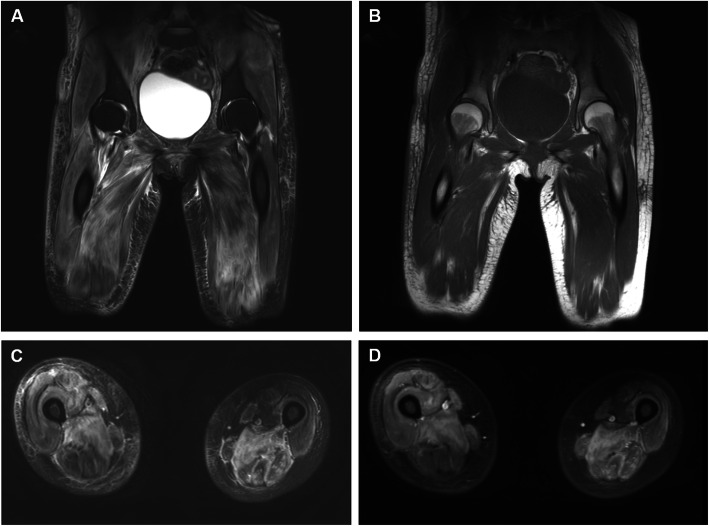
Magnetic resonance imaging of the thigh. Coronal images show **A** increased T2 signal intensity and **B** normal T1 signal intensity in the pelvis and proximal thigh. **C** Axial T2-weighted and **D** gadolinium-enhanced T1-weighted image shows symmetric diffuse muscle edema and inflammation with relative preservation in the left anterior compartment of the thigh

**Fig. 3:**
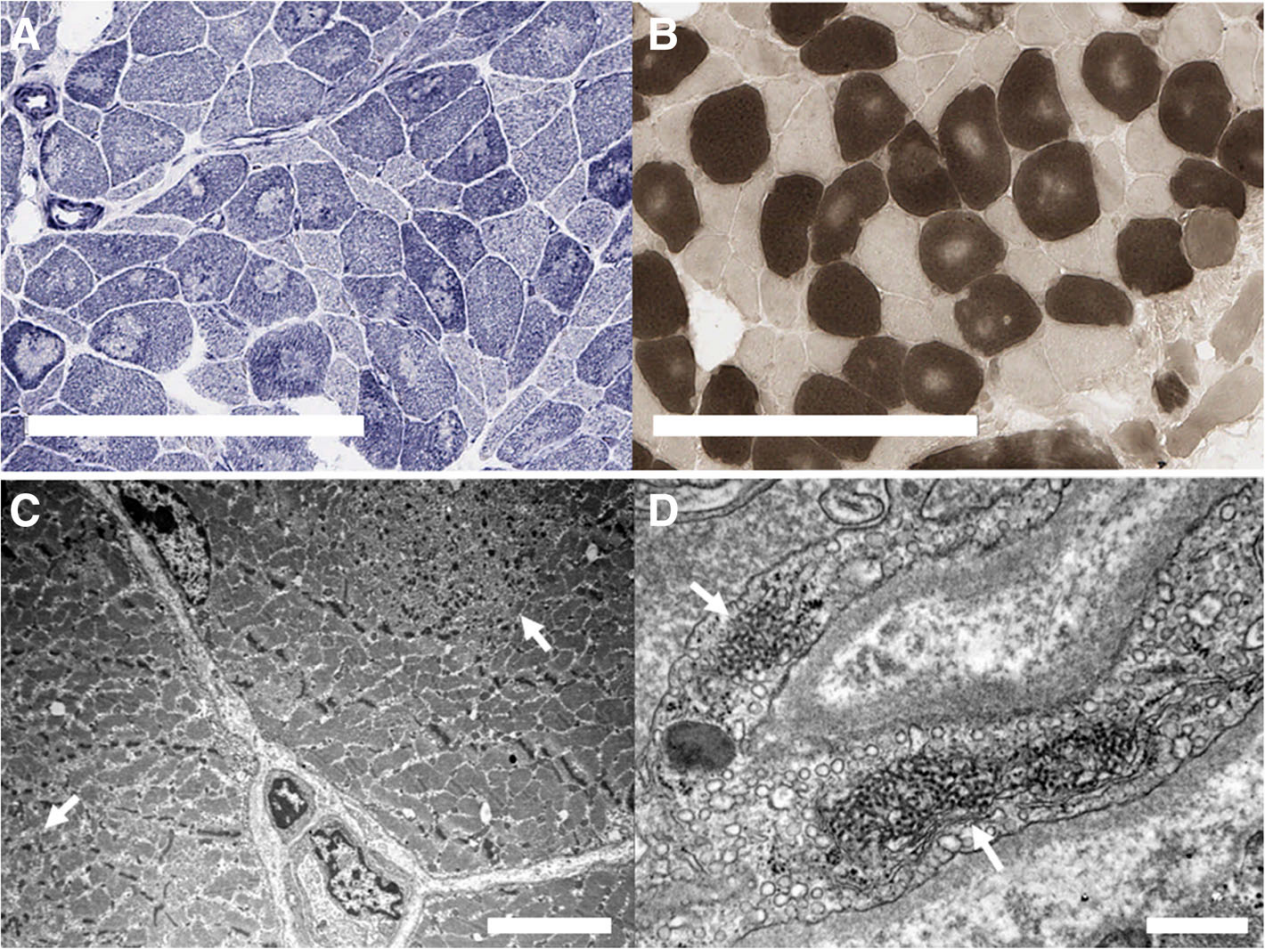
Histological findings in a muscle biopsy specimen obtained from the vastus lateralis. **A** NADH-tetrazolium reductase stain shows irregularly outlined central cores in the type I myofibers (bar: 200 μm). **B** ATPase pH 4.3 shows pale central cores in the type I myofibers (bar: 200 μm). Electron microscopy (adenyl acetate and lead citrate stain) shows **C** a structured central core composed of randomly scattered short Z-bands and fine filaments (arrow) (bar: 5 μm), and **D** two tubuloreticular bodies (arrows) in the endothelial cells (bar: 500 nm)

On physical examination, her vital signs were stable, and her body mass index was 21.8 kg/m^2^ (height 156 cm, weight 53 kg). Her bilateral muscle strength was grade 4/5 at neck flexion, 4/5 at shoulder abduction, 4/5 in wrist flexion, 4/5 in hand grip, 2/5 in the proximal hips, and 4/5 in ankle dorsiflexion. She had normal reflexes of the biceps brachii, patella, and ankle. Facial erythema involving the nasolabial folds and an erythematous rash on the upper back and anterior chest wall compatible with V-neck sign and shawl sign were observed. Skin ulcers were seen on the hand knuckles, right elbow and lateral aspect of the right thigh (Fig. 1). She had widespread, palpable soft tissue thickening of the bilateral thigh and upper arm with tenderness, which turned out to be calcification. The laboratory results were as follows: white blood cell count, 6440/mm^3^ with lymphopenia (lymphocyte 322/mm^3^); haemoglobin, 9.1 g/dL; platelet, 148,000/mm^3^; erythrocyte sedimentation rate (ESR), 72 mm/hr.; C-reactive protein (CRP), 0.44 mg/dL; aspartate aminotransferase (AST), 95 (1–40) IU/L; alanine aminotransferase (ALT), 30 (1–40) IU/L; creatine kinase (CK), 128 (20–270) IU/L; lactate dehydrogenase (LDH), 553 (100–225) g/dL; myoglobin, 47.1 (0–106) ng/mL; aldolase, 10.8 (0–7.6) U/L; C3, 83 (83–193) mg/dL; and C4, 19 (15–57) mg/dL. The antinuclear antibody test was positive and showed a homogeneous pattern at a 1:160 dilution, but the anti-dsDNA, anti-Sm, anti-Ro, anti-La, anti-Scl-70, anti-centromere, anti-U1 ribonucleoprotein, anti-phospholipid, and anti-Jo-1 antibody tests were all negative. Magnetic resonance imaging (MRI) of the thigh showed symmetric diffuse muscle signal changes with an enhanced T2 signal and diffuse patchy contrast enhancement involving both the anterior and posterior compartments of the thigh (Fig. 2). Her radiographs of shoulders and pelvis showed extensive, plaque-like soft tissue calcification of both hips, thighs and upper arms. Electromyography (EMG) showed fibrillations, positive sharp waves (PSWs), increased amplitudes of motor unit action potential (MUAP) and reduced interference pattern in the biceps. In the EMG of the tibialis and gastrocnemius muscles, fibrillations, PSWs, small and polyphasic MUAPs, and reduced interference pattern were identified. In contrast, the nerve conduction study revealed normal responses. A muscle biopsy of the right vastus lateralis demonstrated many degenerating and regenerating muscle fibers and perifascicular atrophy with moderate infiltration of inflammatory cells in the endomysial and perivascular areas, consistent with dermatomyositis. However, enzyme histochemical analysis showed centrally placed cores in the type I myofibers with nicotinamide adenine dinucleotide dehydrogenase (NADH) and succinate dehydrogenase (SDH) staining, suggesting CCD (Fig. 3a). Electron microscopy showed both tubuloreticular bodies in the endothelial cells and disorganized myofilaments with scattered short Z-bands and structured central cores in the muscle fibers, findings that suggested a diagnosis of CCD with superimposed dermatomyositis (Fig. 3c and d). Computed tomography of the chest and abdomen showed no evidence of interstitial lung disease or malignancy. The echocardiography was normal. The patient had no pathogenic mutations in the genes associated with congenital myopathy, including *RYR1, SEPN1, ACTA1, AGRN, BIN1, CFL2, CHAT, CHRNA1, CHRNB1, CHRND, CHRNE, COLQ, DNM2, DOK2, GFPT1, IGHMBP2, KBTBD13, KLHL40, LAMB2, MTM1, MUSK, MYH7, RAPSN, SLC5A7, SMN1, TNNT1, TPM2, TPM3* and *TTN*, in hybridization capture-based next-generation sequencing. However, she had a heterozygous mutation in the *NEB* gene of c.8318G > A (p.Arg2773Gln), which is considered a variant of uncertain significance. Parental genetic testing was negative.

**Fig. 4:**
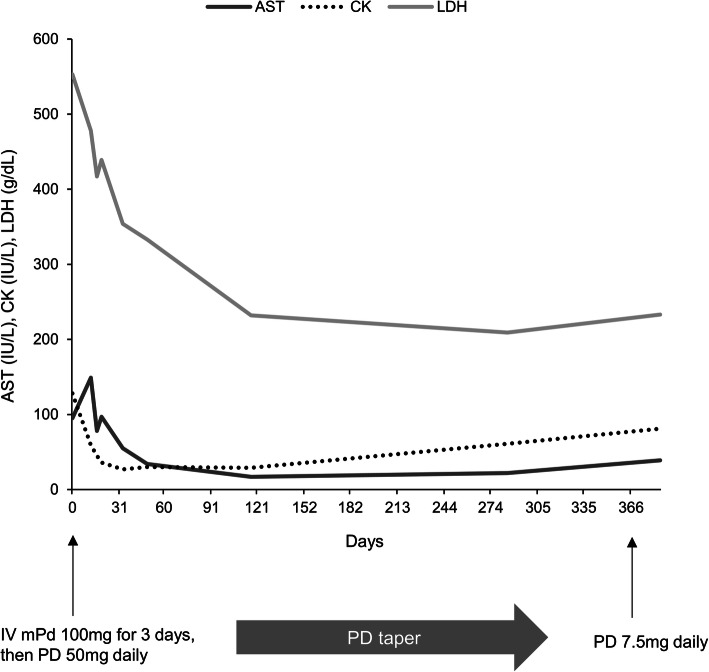
Changes in serum levels of skeletal muscle enzymes and daily doses of glucocorticoid during the follow-up period. AST, aspartate aminotransferase, CK, creatine kinase, LDH, lactate dehydrogenase, PD, prednisolone, mPd, methylprednisolone

The patient received intravenous methylprednisolone at 100 mg daily for 3 days, followed by oral prednisolone at 25 mg twice a day and azathioprine at 50 mg daily. Additionally, she started regular physiotherapy and rehabilitation to maintain mobility. One month later, the patient had a significant improvement in muscle strength with a decrease in muscle enzyme levels (Fig. 4). The muscle strength for hip flexion improved to grade 4/5 after 1 month of treatment and to grade 5/5 (normal) after 3 months of treatment. The patient did not complain of respiratory symptoms and had no abnormal findings on chest radiographs after 1 year of follow-up. Currently, the patient is able to walk on her own, and is taking 7.5 mg/day of prednisolone and 100 mg/day of azathioprine.

## Discussion

Dermatomyositis is an immune-mediated inflammatory muscle disease characterized by proximal muscle weakness. In contrast, CCD shows the disruption of intracellular muscle calcium homeostasis and ECC by genetic mutations, and is associated with typically proximal muscle weakness. We described a patient with severe, debilitating proximal muscle weakness, elevated muscle enzyme levels, myopathic EMG abnormalities, skin rash, and calcinosis who was clinically diagnosed with dermatomyositis. However, her muscle biopsy revealed the characteristic findings of both dermatomyositis and CCD, suggesting that dermatomyositis occurred in this CCD patient who had no previous symptoms. The coexistence of dermatomyositis and CCD may be a coincidence, but it may also suggest a link between autoimmunity and genetic muscle disease that affects intracellular calcium homeostasis.

The congenital myopathies are a group of early-onset, non-dystrophic genetic neuromuscular diseases with characteristic muscle biopsy findings, consisting of CCD, multi-minicore disease (MmD), centronuclear myopathy and nemaline myopathy [2]. Congenital myopathies have been attributed to mutations in genes encoding proteins implicated in skeletal muscle calcium homeostasis, ECC, thin–thick filament assembly, and their interactions [2]. For a muscle contraction to occur, calcium release from the sarcoplasmic reticulum (SR) to the sarcoplasm leads to sarcomere shortening through interactions between the thin and thick filaments driven by adenosine triphosphate (ATP) [7]. This ECC is terminated by calcium reuptake by the SR. Therefore, continuous calcium leak to the sarcoplasm and disturbed ECC causes muscle weakness to develop in congenital myopathies, especially CCD. Histologically, CCD is characterized by centrally located, well-demarcated cores of diminished or absent oxidative enzyme activity in the muscle fibers. Type I fiber predominance and atrophy are common in CCD [8]. CK levels may be normal or mildly elevated. Selective muscle involvement with increased T1 signal seen on muscle MRI depends on the subset of congenital myopathy, which can also be distinguished from inflammatory myopathy [9].

CCD is closely associated with dominant *RYR1* mutations, implying that the typical central cores on muscle biopsy are highly suggestive of *RYR1* defect [2]. Previously, *RYR1* mutations was reported to be responsible for 47–67% of patients with CCD, suggesting that the disease is genetically heterogeneous [10–12]. However, the mutation screening performed in these studies was limited to the three hot spots of the *RYR1* gene, especially the C-terminal region most relevant to CCD. In contrast, a previous study covering the full coding regions of the *RYR1* gene in a cohort of Japanese CCD patients showed that *RYR1* mutations occur in 93% of CCD patients [12]. Of note, functional studies are needed when mutations are detected in the *RYR1* and *NEB* genes which are associated with congenital myopathies, as variants of uncertain significance are common in these genes due to their large size [13]. In addition, mutations in the RYR1-binding proteins, FKBP12 and CACNA1S, identified as directly participating in ECC, may also contribute to the RYR1 channel dysfunction [14]. Mutations have been identified in CACNA1S in patients with malignant hyperthermia and primary periodic paralysis [12, 15]. Thus, RYR1-binding proteins may also need to be studied in more detail in patients with CCD.

Targeted next-generation sequencing did not detect any genetic mutations related to congenital myopathy, including *RYR1*, in our patient. However, the typical central cores were clearly revealed by NADH and SDH staining of a majority of muscle fibers in the patient. In addition, ultrastructually, disarray of the myofilaments with scattered short Z-bands and structured central cores was observed, which is highly suggestive of CCD. Inclusion body myositis can show mitochondrial abnormalities such as cytochrome c oxidase (COX) deficiency in muscle fibers, but COX-negative/SDH-positive staining is seen throughout the muscle fibers rather than in the central region [16]. Histological signs of deficiency in oxidative enzyme activities in dermatomyositis and polymyositis have not been shown in previous reports. Patients with CCD without identifiable *RYR1* mutations may have mutations in unknown genes or *RYR1* mutations may still exist in unexamined regions such as promoter region, introns and casual copy number variations.

Several muscular dystrophies, such as facioscapulohumeral dystrophy and limb-girdle muscular dystrophy, show muscle regeneration and inflammatory cell infiltrates in muscle fibers, similar to inflammatory myopathy [17]. Additionally, myositis-specific autoantibodies such as anti-Jo-1 and anti-Mi2 were found in patients with muscular dystrophy [6]. Based on these findings, autoantigens may be expressed in the regenerating muscle fibers of muscular dystrophy, triggering an autoimmune process and overlapping with inflammatory myopathy [18]. In contrast, congenital myopathies rarely show muscle regeneration, and may lead to very low levels of myositis autoantigen expression. Instead, aberrant intracellular calcium homeostasis in congenital myopathy may affect the immune system. For instance, increased T cell receptor-mediated calcium influx in lupus T cells contributes to T cell activation by inducing the activation of calcineurin [19]. RYR1, a calcium-release channel protein associated with CCD, is expressed preferentially in skeletal muscle, but is also expressed in B cells and dendritic cells. RYR1-mediated intracellular calcium influx in B cells and dendritic cells may cause their activation and the release of pro-inflammatory cytokines [20–22]. Interestingly, dermatomyositis is characterized by abnormal humoral immunity related to B cell activation producing autoantibodies. Previous studies have reported rare cases of muscular dystrophy coexisting with autoantibodies. However, to the best of our knowledge, this is the first report of inflammatory myopathy coexisting with congenital myopathy and the first report of autoantibodies found in the patient with congenital myopathy. Further studies on autoantibodies in CCD patients may be needed when dermatomyositis or other autoimmune disease is suspected in patients with CCD.

The management of congenital myopathies is mainly supportive [23], whereas inflammatory myopathies respond well to treatment with glucocorticoid and immunosuppressive agents. Immunosuppressive therapy was also effective in improving muscle strength in the present case with dermatomyositis coexisting with CCD. Therefore, inflammatory myopathy should be suspected in a patient with congenital myopathy if he or she presents with a sudden deterioration in muscle strength, particularly along with extramuscular symptoms or the presence of autoantibodies. We suggest that a muscle biopsy or MRI should be performed to evaluate for overlapping inflammatory myopathy.

In summary, we reported a rare case of the coexistence of dermatomyositis and CCD. Clinicians should be aware that patients with CCD could have inflammatory myopathy, which responds well to immunosuppressive therapy. Testing for autoantibodies, and muscle MRI or muscle biopsy may help distinguish between inflammatory myopathy and underlying genetic muscle diseases. Further research may be needed to decipher the association between autoimmunity and congenital myopathy-associated intracellular calcium homeostasis.

## Data Availability

The datasets used during the current study are available from the corresponding author on reasonable request.

## Abbreviations

ALT: Alanine transaminase
AST: Aspartate transaminase
ATP: Adenosine triphosphate
CCD: Central core disease
CK: Creatine kinase
COX: Cytochrome c oxidase
CRP: C-reactive protein
ECC: Excitation-contracture coupling
EMG: Electromyography
ESR: Erythrocyte sedimentation rate
LDH: Lactate dehydrogenase
MmD: Multi-minicore disease
MRI: Magnetic resonance imaging
MUAP: Motor unit action potential
NADH: Nicotinamide adenine dinucleotide dehydrogenase
PSW: Positive sharp wave
RYR1: Ryanodine receptor 1
SDH: Succinate dehydrogenase
SR: Sarcoplasmic reticulum
